# RNF187 inhibits porcine reproductive and respiratory syndrome virus replication by recruiting the autophagy receptor NDP52 to degrade nsp12

**DOI:** 10.1186/s13567-026-01824-9

**Published:** 2026-08-01

**Authors:** Qiumei Wang, Guoxin Zheng, Tao Tian, Anli Chen, Guihong Zhang, Heng Wang

**Affiliations:** 1 Guangdong Provincial Key Laboratory of Zoonosis Prevention and Control, College of Veterinary Medicine, South China Agricultural University, Guangzhou, 510642 China; 2 National Engineering Research Center for Breeding Swine Industry, South China Agricultural University, Guangzhou, 510642 China

**Keywords:** Porcine reproductive and respiratory syndrome virus, Nsp12, RNF187, interaction, protein degradation, antiviral

## Abstract

Porcine reproductive and respiratory syndrome is one of the most devastating diseases affecting global swine production. Viral nonstructural protein 12 (nsp12) is a key factor in viral replication during viral subgenomic RNA synthesis. We identified the host E3 ubiquitin ligase RING finger protein 187 (RNF187) as a novel anti-porcine reproductive and respiratory syndrome virus (PRRSV) host-restriction factor. RNF187 directly interacts with nsp12, and the overexpression of RNF187 significantly inhibits the expression of the viral nucleocapsid (N) protein and the titer of viruses, whereas the knockdown of RNF187 promotes viral replication. Mechanistic studies showed that RNF187 mediates the degradation of nsp12 in a dose-dependent manner. This degradation process can be blocked by the autophagy inhibitor, 3-methyladenine (3-MA). These results indicate that RNF187 degrades nsp12 via the autophagy pathway rather than the proteasome pathway. Mechanistically, RNF187 acts as a scaffold protein to recruit the autophagic cargo receptor NDP52 and promotes the K63-linked polyubiquitination at lysine 130 (K130) of nsp12, thereby targeting nsp12 for autophagy-lysosomal degradation. This study revealed a novel host defense mechanism mediated by the RNF187-NDP52 axis, which restricts PRRSV infection by targeting nsp12, thereby providing a potential target for the development of antiviral strategies.

## Introduction

Porcine reproductive and respiratory syndrome is an infectious disease caused by the porcine reproductive and respiratory syndrome virus (PRRSV) and is characterized by reproductive disorders in sows and respiratory symptoms in piglets [1]. The first PRRSV outbreak occurred in the late 1980s. In 2006, an outbreak of “high fever disease” caused by PRRSV occurred in China. Compared to traditional strains, the nsp2 protein of the epidemic strain had a deletion of 30 amino acids. This disease has become one of the most destructive diseases in the Chinese pig farming industry, causing significant economic losses [2]. PRRSV is an enveloped single-stranded positive-sense RNA virus. Its genome encodes multiple structural (GP2-N protein) and nonstructural (nsp1–nsp12) proteins [3,4]. Nonstructural proteins play important roles in viral RNA synthesis, protease activity, replication-associated membrane rearrangement, virulence determination, and the regulation of host immune responses [5].

Nsp12 is a viral protein composed of 153 amino acid residues with a highly conserved sequence among different PRRSV strains. The N-terminus of this protein contains two membrane-targeting regions that are primarily localized in the cytoplasm and have a punctate distribution [6]. As a key core factor of the viral replication complex, nsp12 interacts with multiple nonstructural proteins, including nsp1α, nsp1β, nsp2, nsp3, nsp5, nsp6, nsp9, nsp10, and nsp11 [7]. Meanwhile, under oxidative conditions, nsp12 can form dimers and specifically participates in the synthesis of positive and negative strands of viral subgenomic mRNA, but not in the replication process of genomic RNA. The C35 and C79 sites are crucial for their function. The helicase nsp10 can regulate its interaction with nsp12 through an intramolecular switch, thereby affecting the synthesis of subgenomic RNA [8].

Additionally, nsp12 regulates viral replication by interacting with multiple host proteins. For example, heat shock protein 70 (HSP70) can bind to nsp12 and maintain its protein stability, thereby promoting viral replication, whereas RING finger protein 114 (RNF114), galectin-3, and proteasome subunit beta type-1 (PSMB1) exert antiviral effects by mediating the ubiquitination or autophagic degradation of nsp12 [9–12]. Meanwhile, nsp12 can induce signal transducer and activator of transcription 1 (STAT1) phosphorylation and upregulate the expression of pro-inflammatory cytokines and chemokines such as interleukin (IL)-1β, IL-8, C–C motif chemokine ligand 2 (CCL2), and C-X-C motif chemokine ligand 10 (CXCL10), participating in the pathogenic process of PRRSV [13,14]. Therefore, elucidating the molecular mechanisms of host–nsp12 interactions and identifying novel host targets to inhibit viral replication are of great theoretical and practical significance for antiviral intervention.

RNF187, also known as RING domain AP-1 co-activator-1 (RACO-1), is an E3 ubiquitin ligase containing a RING finger structure. The N-terminal RING finger domain is responsible for recruiting E2 ubiquitin-conjugating enzymes and catalyzing substrate ubiquitination, whereas the C-terminus of RNF187 mainly mediates its interactions with various substrate proteins [15]. Functionally, RNF187 is involved in multiple biological processes, including cell proliferation, migration, and DNA damage repair. Studies have shown that RNF187 can regulate the signaling pathway of p53 and NF-κB by mediating its ubiquitination and degradation [16], and it is a key factor through which Notch1 promotes hepatocellular carcinoma (HCC) invasion and metastasis [17]. However, its role in viral infections has been studied less extensively.

This study investigated the regulatory relationship between RNF187 and PRRSV replication, and confirmed that RNF187 interacts with viral nsp12 and mediates its degradation. Using yeast two-hybrid screening and co-immunoprecipitation, we demonstrated that the host protein RNF187 can specifically bind to nsp12. Furthermore, RNF187 mediates the degradation of nsp12 via the autophagy pathway, thereby inhibiting PRRSV replication. This study systematically elucidated the molecular mechanism by which the novel antiviral factor RNF187 regulates PRRSV replication, providing new insights into host defense against PRRSV infection.

## Materials and methods

### Cells culture and virus

HEK-293T and Marc-145 cells were supplied by our laboratory and maintained in fresh Dulbecco’s Modified Eagle Medium (DMEM; Gibco, C11995500) supplemented with 10% fetal bovine serum (FBS; VivaCell Biosciences, 2238253) at 37 °C under 5% CO_2_. The PRRSV XH-GD strain was obtained from our laboratory.

### Antibodies and reagents

Anti-HA antibody (AF2305) was purchased from Beyotime (Shanghai, China). Anti-Flag (M2008L) and anti-GFP-tagged mAb magnetic beads (M20016M) were purchased from Abmart (Shanghai, China). The anti-Flag magnetic beads (B26102), 3-MA (S2767), MG132 (S2619), and Z-VAD-FMK (S7023) were purchased from Selleck (Shanghai, China). Anti-RNF187 antibody (NBP2-83456) was purchased from Novus (Centennial, USA). The PRRSV anti-N-protein antibodies were maintained in the laboratory.

### Yeast two-hybrid library to identify host proteins that interact with nsp12

The porcine alveolar macrophage (PAM) cDNA library was constructed in the laboratory, and host proteins interacting with PRRSV nsp12 were screened using the yeast two-hybrid (Y2H) technique. Viral nsp12 was inserted into the pGBKT7 vector to construct the bait plasmid pGBKT7-nsp12. Bait auto-activation and toxicity tests were also performed. The bait plasmid and empty prey vector were co-transformed into Y2H Gold competent cells. Growth on double dropout (DDO, SD/-Leu/-Trp) plates indicated the successful transfer of the recombinant bait plasmid into the host bacteria and the absence of toxicity to the host bacteria. The lack of growth on triple dropout (TDO, SD/-His/-Leu/-Trp) plates indicated that the bait plasmid could not activate the yeast reporter gene HIS3. The lack of growth on the quadruple dropout (QDO, SD/-Trp/-Leu/-His/-Ade) plates indicated that the bait plasmid could not activate the expression of the yeast reporter gene ADE2, allowing progression to the next step of library screening. The library plasmids were transformed into competent bait strain cells. The positive clones were subjected to selective pressure screening and colony polymerase chain reaction (PCR). Sequencing and alignment were performed to screen for potential interacting proteins.

### Plasmid construction and transfection

The full-length coding sequence of porcine RNF187 was amplified from porcine cDNA by PCR using gene-specific primers. The amplified target fragment was purified, double-digested with corresponding restriction endonucleases, and subsequently ligated into the eukaryotic expression vectors carrying HA and mCherry tags, to generate recombinant plasmids with C-terminal tags. Similarly, the complete coding region of PRRSV nsp12 gene was amplified from viral cDNA template, followed by restriction enzyme digestion and ligation into Flag- and EGFP-tagged expression vectors to generate recombinant plasmids with C-terminal tags. Marc-145 cells were transfected with Lipofectamine 3000 (Thermo Fisher Scientific, CA, USA), and 293T cells were transfected with polyethylenimine (PEI) reagent (FuShen, China). Lipofectamine 3000 or PEI reagent was used to transfect expression following the manufacturer’s protocol.

### RNA interference

The small interfering RNA (siRNA) was designed to knock down RNF187 expression in Marc-145 cells and was diluted with enzyme-free water. The siRNA was transfected into Marc-145 cells using the ReFect transfection reagent (Bai Dai, China) following the manufacturer’s protocol. The siRNA sequences are listed in Table 1.

**Table 1:** **The siRNA sequence for RNF187**

Primer name	Sequence (5′-3′)
Monkey RNF187-F	ACAAGGGGTCTGTGGAAATCAT
Monkey RNF187-R	TCCATCACGTGTCCCTTCCAC
Monkey GAPDH-F	TGATGACATCAAGAAGGTGGTGAAG
Monkey GAPDH-R	TCCTTGGAGGCCATGTGGGCCAT

### Western blot assay

The cells were washed three times with cold phosphate-buffered saline (PBS), and subsequently lysed in NP40 lysis buffer containing phenylmethylsulfonyl fluoride (PMSF) (Beyotime, China). Cells were collected and separated by sodium dodecyl sulfate–polyacrylamide gel electrophoresis (SDS–PAGE), after which the samples were transferred to a 0.45-µm nitrocellulose membrane (Millipore, USA). The membrane was blocked with 5% milk (Yili, China) at room temperature for 2 h and then incubated with the corresponding primary antibodies. After overnight incubation and three washes with Tris-buffered saline with Tween 20 (TBST), the membrane was incubated with a secondary antibody at room temperature for 1 h. Protein detection was performed using the Sapphire system.

### Immunoprecipitation assay

For anti-Flag/HA/enhanced green fluorescent protein (EGFP) immunoprecipitation assay, HEK-293T cells were cultured in 60-mm-diameter dishes and transfected plasmids. After 36 h of culture, the cells were washed twice with PBS and lysed on ice using western blot/immunoprecipitation (WB/IP) lysis buffer supplemented with PMSF. Lysates were collected and centrifuged, and the protein samples were incubated with 30 µL of Flag/HA/EGFP-tagged antibody beads at 4 °C for 8 h with gentle stirring. Meanwhile, concurrently, protein samples were incubated with 30 µL of IgG antibody beads at 4 °C for 8 h to serve as the control group. The beads were subsequently washed with 800 µL of ice-cold PBS with Tween 20 (PBST), and 50 μL of 1× SDS loading buffer was added, followed by heating for protein denaturation and subsequent western blot detection.

### Immunofluorescence assay

Once the cell growth stabilized, the plasmids were transfected. After 24 h, cells were washed with cold PBS and fixed with 4% paraformaldehyde. Cells were then washed with cold PBS and permeabilized with 0.1% Triton X-100, blocked with 5% bovine serum albumin (BSA), and incubated with the appropriate primary antibodies overnight. After three washes with PBS, secondary antibodies (1:1000 dilution) were added and incubated at room temperature for 1 h. DAPI was added to each well, and the cells were incubated for 10 min. Images were captured using a laser-scanning confocal microscope. Image co-localization was analyzed using ImageJ software.

### Quantitative real-time PCR (RT-qPCR) assay

Total RNA was extracted and isolated using an RNA extraction kit (Fastagen, China) and reverse transcribed into cDNA using a reverse transcription enzyme (Vazyme, China). RT-qPCR was conducted according to the manufacturer’s protocol using the ChamQ Universal SYBR qPCR Master Mix on a CFX96 real-time system (Bio-Rad, USA). The mRNA expression levels were quantified via the 2^−ΔΔCT^ method. The qPCR primer sequences are listed in Table 2.

**Table 2:** **Primer sequence for RT-qPCR**

Primer name	Sequence (5′-3′)
Monkey RNF187-F	ACAAGGGGTCTGTGGAAATCAT
Monkey RNF187-R	TCCATCACGTGTCCCTTCCAC
Monkey GAPDH-F	TGATGACATCAAGAAGGTGGTGAAG
Monkey GAPDH-R	TCCTTGGAGGCCATGTGGGCCAT

### Statistical analysis

All data were presented as means ± standard deviation (SD). Statistical analysis was conducted using Student’s *t*-test with GraphPad Prism 8.0. *P* < 0.05 was considered statistically significant, ns means no significant difference, *P < 0.05, **P < 0.01, ***P < 0.001.

## Results

### RNF187 interacts with nsp12

**Figure 1:**
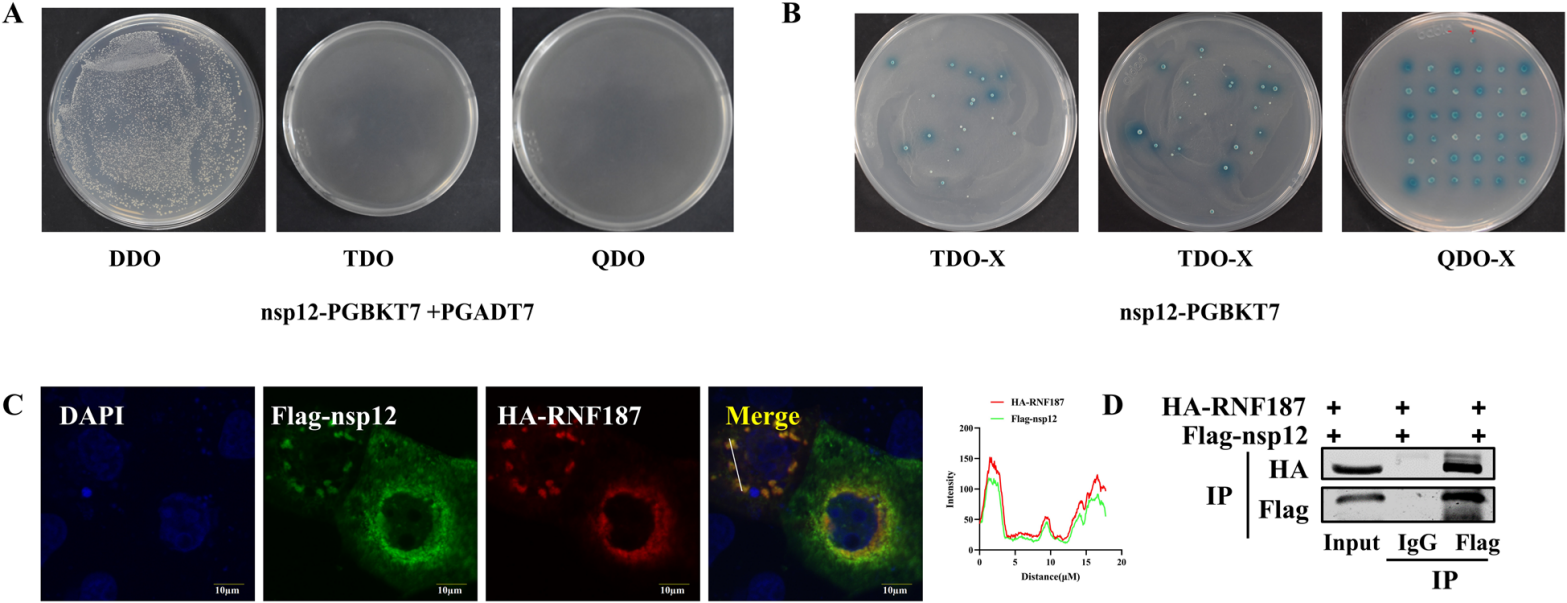
**Interaction between RNF187 and nsp12.** **A** Y2H Gold competent cells were co-transformed with the bait plasmid pGBKT7-nsp12 and the prey empty vector pGBKT7. Subsequently, cells were plated on DDO, TDO, and QDO plates to perform nsp12-PGBKT7 bait auto-activation detection and toxicity testing. **B** Plate yeast cells were transformed with the nsp12-PGBKT7 bait on TDO/X plates. Then, the transformed yeast cells were spotted onto QDO/X plates (+: yeast cells co-transformed with PGBKT7-53 and PGADT7-T; −: yeast cells co-transformed with PGBKT7-lam and PGADT7-T). **C** HA-RNF187 and Flag-nsp12 were co-transfected into Marc-145 cells. After 24 h, the cells were fixed and stained. Co-localization analysis was performed using ImageJ. **D** HA-RNF187 and Flag-nsp12 were co-transfected into 293T cells. After 36 h, the supernatant was removed, and immunoprecipitation was performed using Flag-specific magnetic beads.

Nsp12, as a key protein in PRRSV genomic RNA synthesis, plays a crucial role in the viral replication cycle [18]. To further elucidate the biological functions of nsp12 and its interaction network with host proteins, we constructed a PAM cDNA library and screened for host factors that interact with nsp12 using a yeast two-hybrid system with nsp12 as the bait protein. Prior to formal screening, self-activation of the pGBKT7-nsp12 bait plasmid was detected. Yeast cells transformed with pGBKT7-nsp12 grew and formed white colonies on DDO medium, but were unable to survive on TDO and QDO media, indicating that the bait plasmid lacked autonomous transcriptional activation activity and was suitable for subsequent library screening (Figure 1A). After co-transformation of the cDNA library plasmid with the nsp12 bait plasmid into yeast cells, positive clones were selected from the TDO/X selection plates and further verified by inoculation onto the QDO/X plates. The clones that formed blue colonies were identified as positive interactors (Figure 1B). Through sequence alignment and bioinformatics analysis, 27 host proteins that potentially interacted specifically with nsp12 were obtained (Table 3). Therefore, RNF187 was used for further experiments. After co-transfection of nsp12 and RNF187 eukaryotic expression plasmids into cells, laser confocal microscopy revealed that nsp12 and RNF187 exhibited a dot-like co-localization in the cytoplasm (Figure 1C). Immunoprecipitation experiments confirmed that RNF187 specifically interacted with nsp12 (Figure 1D). These results indicate that RNF187 interacts with nsp12.

**Table 3:** **Host cellular proteins that interact with the PRRSV nsp12 protein as determined by yeast two-hybrid system**

Protein	NCBI ID	Protein	NCBI ID
Mitochondrial import inner membrane translocase subunit TIM14 (DNAJC19)	NP_001177133.1	Heterogeneous nuclear ribonucleoprotein F (HNRNPF)	XP_020929553.1
Toll-interacting protein (TOLLIP)	NP_001302729.1	Glutamine synthetase (GLUL)	NP_999074.1
Probable ATP-dependent RNA helicase DDX5 (DDX5)	XP_020922417.1	Acetolactate synthase-like protein isoform X1 (ILVBL)	XP_005652963.1
Galectin-1	ABE01403.1	Caspase-2	XP_005657777.1
Small ribosomal subunit protein RACK1 (RACK1)	NM_214332.1	Dicarboxylate carrier UCP2(UCP2)	NP_999454.1
Proteasome subunit beta type-7 (PSMB7)	ABK55647.1	Tenascin-X (TNXB)	NP_001116676.1
Annexin A5 (ANXA5)	XP_003129266.2	Plastin-2 (LCP1)	XP_001929173.1
Macrophage migration inhibitory factor (RPSA)	NP_001032223.1	Beta-hexosaminidase subunit beta (HEXB)	NP_999086.2
macrophage migration inhibitory factor (MIF)	NP_001070681.1	Ubiquitin-conjugating enzyme E2 T (UBE2T)	XP_003130657.1
Cathepsin B (CTSB)	NP_001090927.1	Ferritin (FTH1)	NP_999140.1
Transcobalamin-1 (TCN1)	AID16307.1	Esterase D (ESD)	AAG17630.1
Large proline-rich protein BAG6 (BAG6)	NP_001431433.1	Ring finger protein 187 (RNF187)	NP_001422346.1
B9 domain-containing protein 2 (B9D2)	XP_003127202.2		

### The N-terminal domain of RNF187 interacts with the C-terminal domain of nsp12

**Figure 2:**
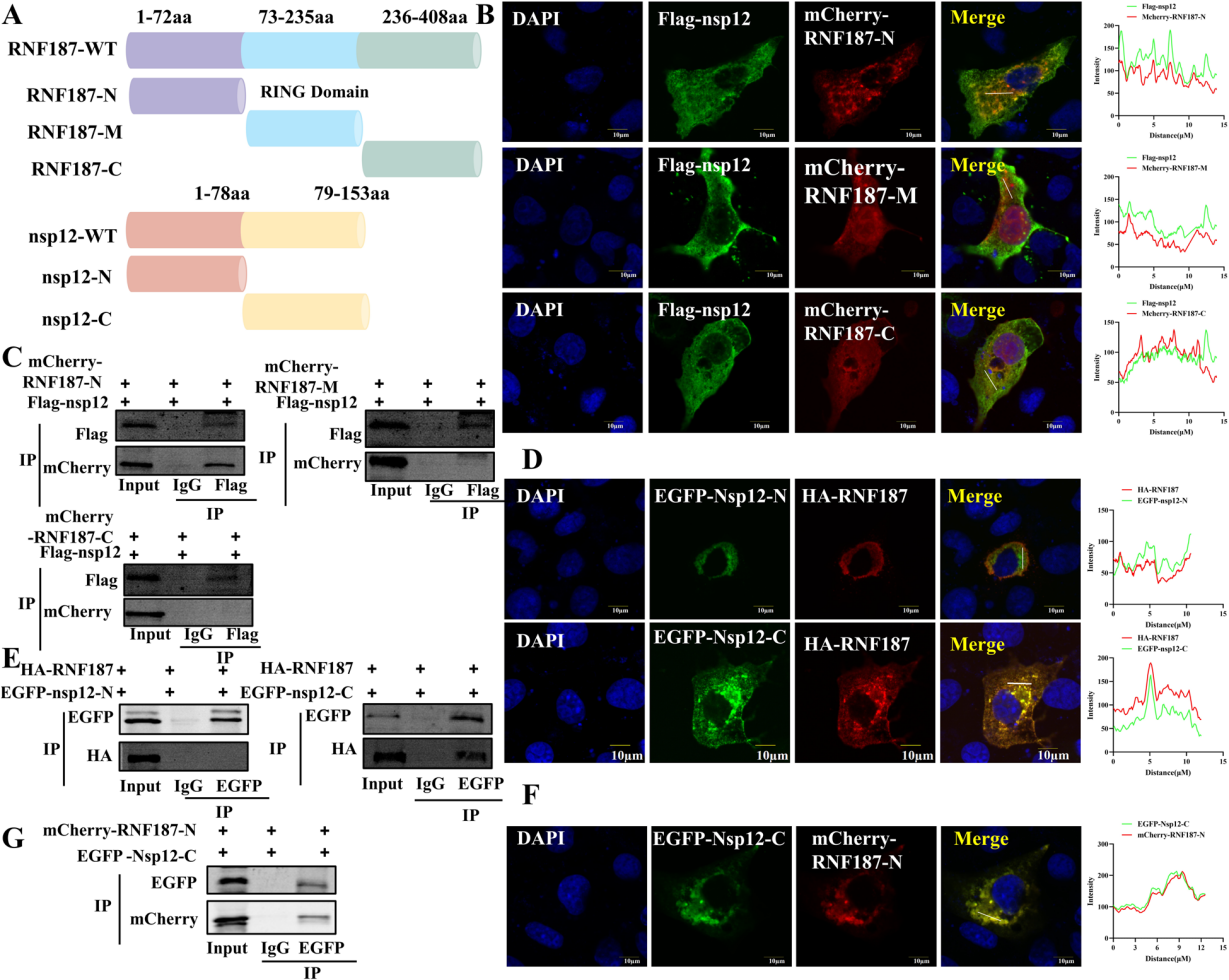
**The N-terminal domain of RNF187 interacts with the C-terminal domain of nsp12.** **A** Schematic diagram of plasmid truncation of RNF187 and nsp12. **B** Flag-nsp12 and mCherry-RNF187 truncation plasmids were co-transfected into Marc-145 cells. After 24 h, the cells were fixed and stained. Co-localization analysis was performed using ImageJ. **C** Flag-nsp12 and mCherry-RNF187 truncation plasmids were co-transfected into 293T cells. After 36 h, the supernatant was removed, and immunoprecipitation was performed using Flag-specific magnetic beads. **D** Co-transfection of cells with EGFP-nsp12 truncation and HA-RNF187 plasmids into Marc-145 cells. After 24 h, the cells were fixed and stained. Co-localization analysis was performed using ImageJ. **E** EGFP-nsp12 truncation and HA-RNF187 plasmids were co-transfected into 293T cells. After 36 h, the supernatant was removed, and immunoprecipitation was performed using EGFP-specific magnetic beads. **F** Co-transfection of cells with EGFP-nsp12 truncation and mCherry-RNF187 truncation plasmids into Marc-145 cells. After 24 h, the cells were fixed and stained. Co-localization analysis was performed using ImageJ. **G** EGFP-nsp12 truncation and mCherry-RNF187 truncation plasmids were co-transfected into 293T cells. After 36 h, the supernatant was removed, and immunoprecipitation was performed using EGFP-specific magnetic beads.

To identify the key structural domains involved in the interaction between RNF187 and nsp12, a series of mCherry-RNF187 and EGFP-nsp12 truncation plasmids (Figure 2A) were constructed. Co-transfection of cells with Flag-nsp12 and mCherry-RNF187 truncation plasmids, followed by laser confocal microscopy, revealed co-localization of nsp12 with the N-terminal domain of RNF187 in the cytoplasm (Figure 2B). Subsequently, 293T cells were co-transfected with Flag-nsp12 and mCherry-RNF187 truncation plasmids, and immunoprecipitation assays demonstrated specific binding between nsp12 and the N-terminal domain of RNF187 (Figure 2C). Co-transfection of cells with EGFP-nsp12 truncation and HA-RNF187 plasmids, followed by laser confocal microscopy, revealed co-localization of the C-terminal domain of nsp12 with RNF187 in the cytoplasm (Figure 2D). Subsequently, 293T cells were co-transfected with the EGFP-nsp12 truncation mutant and HA-RNF187 plasmids, and immunoprecipitation assays demonstrated specific binding between the C-terminal domain of nsp12 and RNF187 (Figure 2E). Finally, co-transfection of cells with EGFP-nsp12-C truncation and mCherry-RNF187-N truncation plasmids, followed by laser confocal microscopy, revealed co-localization of the C-terminal domain of nsp12 with the N-terminal domain of RNF187 in the cytoplasm (Figure 2F). Subsequently, 293T cells were co-transfected with the EGFP-nsp12 truncation mutant and mCherry-RNF187-N plasmids, and immunoprecipitation assays demonstrated specific binding between the C-terminal domain of nsp12 and the N-terminal domain of RNF187 (Figure 2G). These results demonstrated that the N-terminal domain of RNF187 interacts with the C-terminal domain of nsp12.

### RNF187 inhibits PRRSV replication

**Figure 3:**
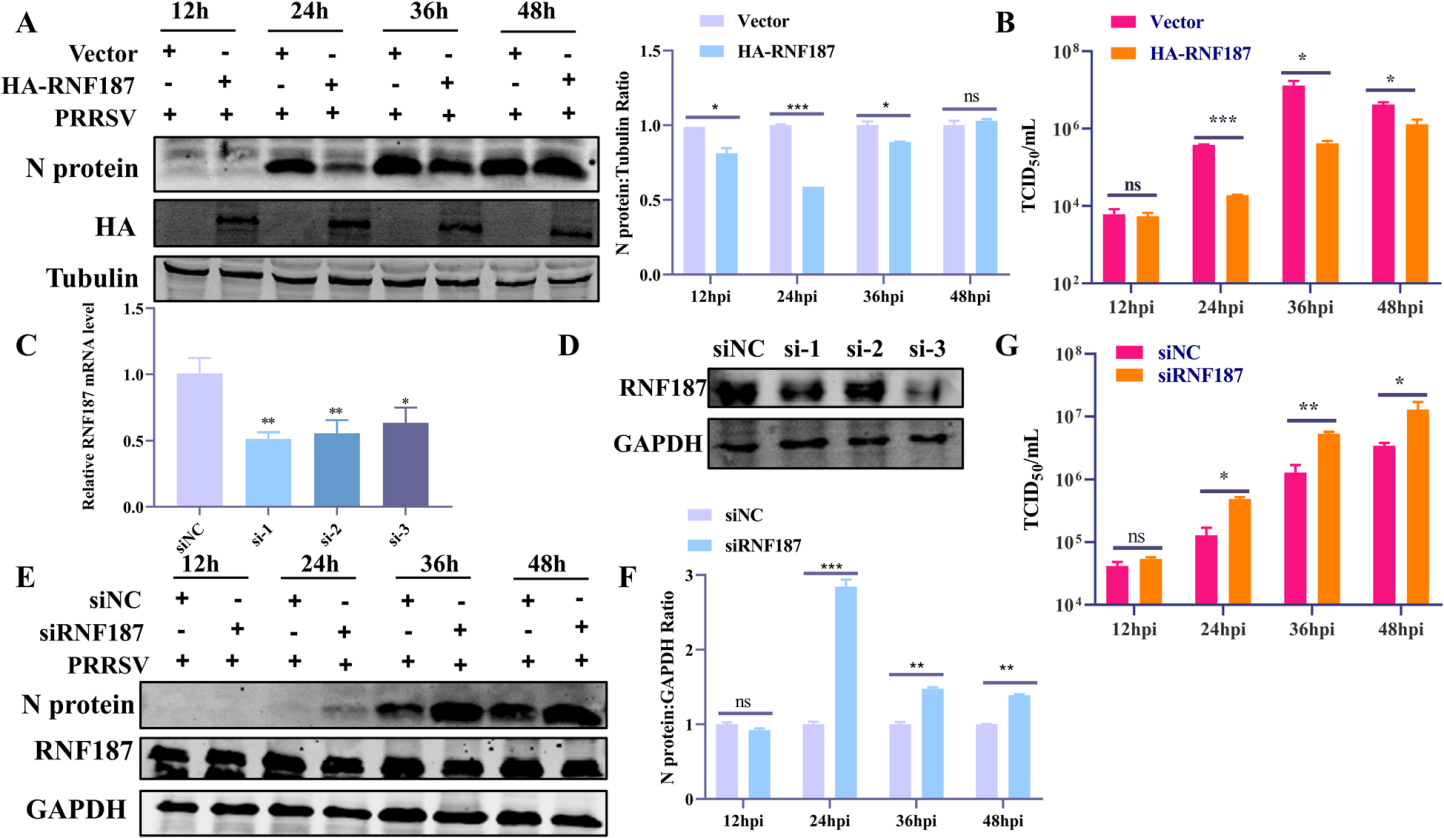
**RNF187 inhibits PRRSV replication. Marc-145 cells were transfected with the HA-RNF187 plasmid.** After transfection for 24 h, PRRSV was inoculated, followed by collection of cells and supernatants. **A** Protein expression of each protein was detected by western blot, with grayscale quantification performed using ImageJ. **B** Viral titers were determined by TCID_50_. Marc-145 cells were transfected with siNC/siRNF187. After transfection for 24 h, cells were collected. **C** RNF187 mRNA expression was detected by RT-qPCR. **D** RNF187 protein expression was detected by western blot. Marc-145 cells were transfected with siNC/siRNF187, after transfection for 24 h, PRRSV was inoculated, followed by collection of cells and supernatants. **E**, **F** Protein expression of each protein was detected by western blot, with grayscale quantification performed using ImageJ. **G** Viral titers were determined by TCID_50_.

To investigate the regulatory role of RNF187 in PRRSV replication, Marc-145 cells were transfected with an RNF187 expression plasmid. After 24 h, the cells were infected with PRRSV (multiplicity of infection [MOI] = 0.5). The cells and supernatants were collected at different time points to detect the expression levels of viral N protein and viral 50% tissue culture infectious dose (TCID_50_). Compared to the control group, RNF187 overexpression significantly inhibited the expression of the viral N protein (Figure 3A) and reduced the titer of the virus (Figure 3B). In contrast, three siRNAs targeting RNF187 were designed and synthesized for knockdown assays. RT-qPCR (Figure 3C) and western blot (Figure 3D) results indicated that siRNF187-1 had the highest silencing efficiency for RNF187 and was used for subsequent experiments. Transfection of siRNF187-1 into Marc 145 cells was followed by PRRSV infection (MOI = 0.1), and samples were collected at the corresponding time points for detection. The knockdown of endogenous RNF187 significantly upregulated the expression level of the viral N protein (Figure 3E and F) and increased the titer of the virus (Figure 3G). Therefore, our findings indicate that RNF187 negatively regulates PRRSV replication.

### RNF187 degrades nsp12 through the autophagy pathway

**Figure 4:**
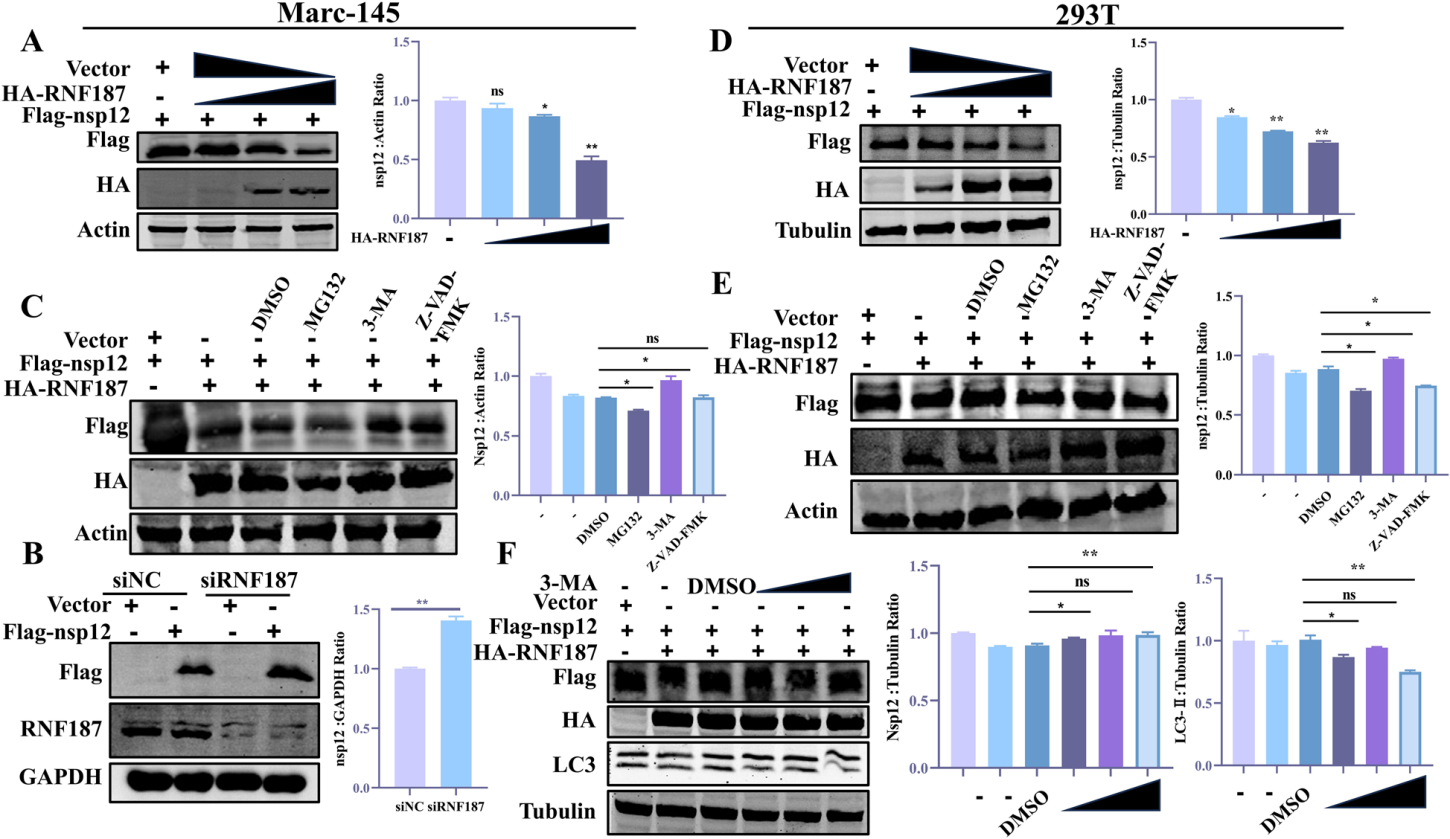
**RNF187 degrades nsp12 through the autophagy pathway.** **A** Marc-145 cells were transfected with HA-RNF187 and Flag-nsp12 plasmid. After transfection for 36 h, cells were collected. Protein expression of each protein was detected by western blot, with grayscale quantification performed using ImageJ. **B** Marc-145 cells were transfected with si-RNF187. After transfection for 24 h, Flag-nsp12 plasmid was transfected into Marc-145 cells, and cells were collected. Protein expression of each protein was detected by western blot, with grayscale quantification performed using ImageJ. **C** Marc-145 cells were transfected with HA-RNF187 and Flag-nsp12 plasmid; after transfection for 24 h, inhibitors MG132, 3-MA, and Z-VAD-FMK were added into cells. Protein expression of each protein was detected by western blot, with grayscale quantification performed using ImageJ. **D** HEK-293T cells were transfected with HA-RNF187 and Flag-nsp12 plasmid. After transfection for 36 h, cells were collected. Protein expression of each protein was detected by western blot, with grayscale quantification performed using ImageJ. **E** HEK-293T cells were transfected with HA-RNF187 and Flag-nsp12 plasmid. After transfection for 24 h, inhibitors MG132, 3-MA, and Z-VAD-FMK were added into cells. Protein expression of each protein was detected by western blot, with grayscale quantification performed using ImageJ. **F** HEK-293T cells were transfected with HA-RNF187 and Flag-nsp12 plasmid. After transfection for 24 h, different concentrations of inhibitor 3-MA were added into cells. Protein expression of each protein was detected by western blot, with grayscale quantification performed using ImageJ.

To further elucidate the molecular mechanism by which RNF187 inhibits PRRSV replication, based on the previous experimental results indicating an interaction between RNF187 and nsp12, we examined the regulation of nsp12 protein levels by RNF187. First, gradient doses of HA-RNF187 and Flag-nsp12 were co-transfected into Marc-145 cells, after which western blot analysis was performed. RNF187 downregulated nsp12 protein expression in a dose-dependent manner (Figure 4A). Similar results were observed in 293T cells (Figure 4D). In contrast, RNF187 was pre-silenced in Marc-145 cells prior to transfection with the Flag-nsp12 plasmid. Knockdown of endogenous RNF187 significantly reversed the degradation of nsp12 protein (Figure 4B). To identify the specific pathway mediated by RNF187 in nsp12 degradation, HA-RNF187 and Flag-nsp12 were co-transfected into Marc-145 cells, which were subsequently treated with the proteasome inhibitor MG132, autophagy inhibitor 3-MA, and apoptosis inhibitor Z-VAD-FMK. 3-MA blocked RNF187-mediated nsp12 degradation, whereas MG132 and Z-VAD-FMK had no obvious effects (Figure 4C). Similar results were obtained in 293T cells (Figure 4E). Finally, HA-RNF187 and Flag-nsp12 were co-transfected into 293T cells, and inhibitors at different concentrations of 3-MA were added to detect the expression levels of nsp12 and LC3-Ⅱ. With an increase in 3-MA concentration, the expression of nsp12 was restored, whereas that of LC3-Ⅱ gradually decreased (Figure 4F). These results confirmed that RNF187 mediates the degradation of nsp12 via the autophagy pathway.

### RNF187 recruits the autophagy receptor NDP52 to degrade nsp12

**Figure 5:**
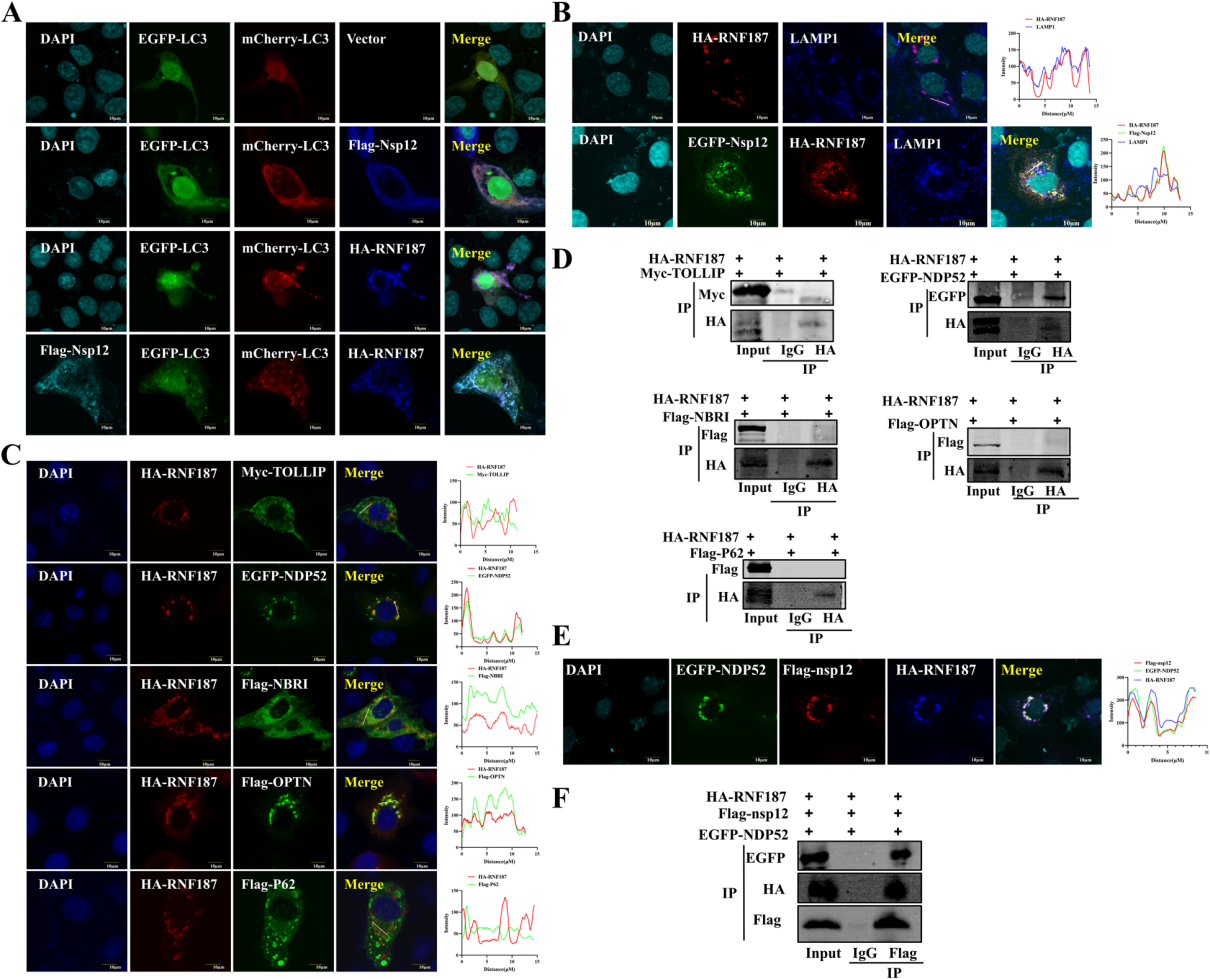
**RNF187 recruits the autophagy receptor NDP52 to degrade nsp12.** **A** Co-transfection of cells with mCherry-GFP-LC3, Flag-nsp12, and HA-RNF187 plasmids into Marc-145 cells. After 24 h, the cells were fixed and stained. **B** Co-transfection of cells with EGFP-nsp12 and HA-RNF187 plasmids into Marc-145 cells. After 24 h, the cells were fixed and stained; co-localization analysis was performed using ImageJ. **C** Co-transfection of cells with P62, NBR1, NDP52, TOLLIP, and OPTN as well as HA-RNF187 plasmids into Marc-145 cells. After 24 h, the cells were fixed and stained; co-localization analysis was performed using ImageJ. **D** Co-transfection of cells with P62, NBR1, NDP52, TOLLIP, and OPTN as well as HA-RNF187 plasmids into 293T cells. After 36 h, the supernatant was removed, and immunoprecipitation was performed using HA-specific magnetic beads. **E** Co-transfection of cells with NDP52, and nsp12 as well as HA-RNF187 plasmids into Marc-145 cells. After 24 h, the cells were fixed and stained; co-localization analysis was performed using ImageJ. **F** Co-transfection of cells with NDP52 and nsp12 as well as HA-RNF187 plasmids into 293T cells. After 36 h, the supernatant was removed, and immunoprecipitation was performed using Flag-specific magnetic beads.

To clarify whether RNF187 induces autophagic flux, the mCherry-GFP-LC3 dual-fluorescent tandem fluorescence plasmid was used. Because GFP fluorescence is quenched in acidic lysosomal environments, whereas mCherry fluorescence remains stable, autophagosomes that fuse with lysosomes to form autolysosomes exhibit a typical complete autophagy phenotype, characterized by reduced green fluorescence and enhanced red fluorescence. Co-transfection of HA-RNF187 with mCherry-GFP-LC3 plasmid into Marc-145 cells was followed by laser confocal microscopy. Compared to the control group, overexpression of RNF187 significantly reduced the number of GFP-positive green fluorescent spots and markedly increased the number of mCherry-positive red fluorescent spots (Figure 5A), indicating that RNF187 can induce complete autophagic flux. However, overexpression of Flag-nsp12 alone did not significantly alter red fluorescence intensity (Figure 5A), suggesting that nsp12 itself does not directly induce autophagy. Additionally, co-localization analysis revealed that both Flag-nsp12 and HA-RNF187 exhibited punctate co-localization with mCherry-LC3 (Figure 5A). To further validate the co-localization of RNF187 and nsp12 in lysosomes, HA-RNF187 was co-transfected with EGFP-nsp12 into Marc-145 cells, and immunofluorescence staining was performed using the lysosomal marker protein LAMP1. Both EGFP-nsp12 and HA-RNF187 exhibited yellow punctate co-localization with LAMP1 (Figure 5B). These findings confirmed that RNF187 mediates nsp12 degradation by inducing complete autophagic flux. To identify the autophagy cargo receptor by which RNF187 degrades nsp12, we examined the interactions between RNF187, multiple autophagy receptors (P62, NDP52, TOLLIP, NBR1, and OPTN), and nsp12. Laser confocal microscopy revealed significant punctate co-localization between RNF187 and the autophagy receptor NDP52 (Figure 5C), and co-immunoprecipitation experiments further confirmed that RNF187 specifically binds to NDP52 (Figure 5D). Subsequently, confocal microscopy results revealed that RNF187 exhibited obvious co-localization with nsp12 and NDP52 in the cytoplasm (Figure 5E). Furthermore, co-immunoprecipitation results confirmed that RNF187 could directly interact with both nsp12 and NDP52 simultaneously (Figure F). We demonstrated that NDP52, a key autophagy cargo receptor, participates in the autophagic degradation of nsp12 mediated by RNF187.

### RNF187 targets the K130 site of nsp12 for K63 ubiquitination

**Figure 6:**
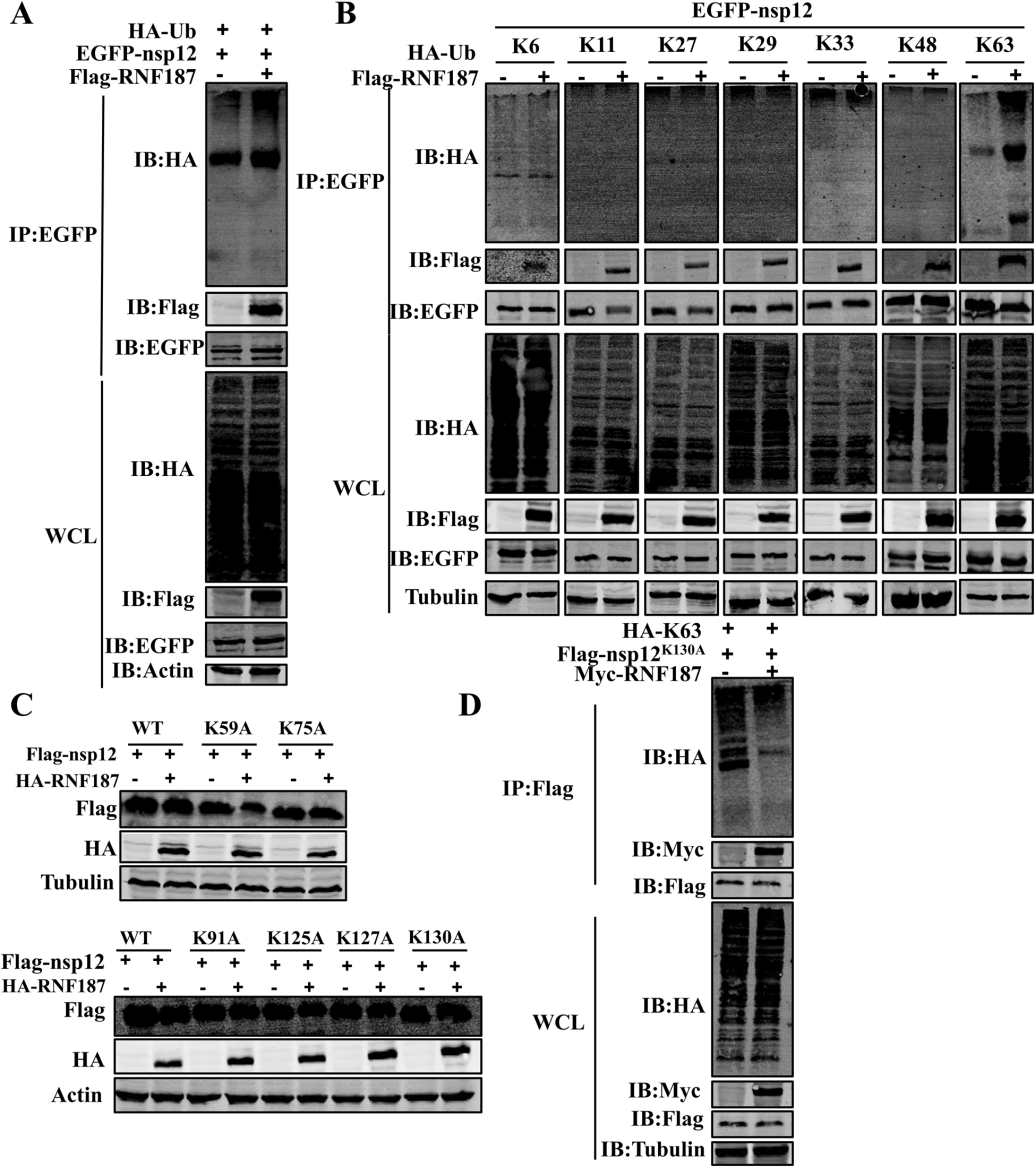
**RNF187 targets the K130 site of nsp12 for K63 ubiquitination.** **A** Co-transfection of cells with HA-Ub, Flag-RNF187, and EGFP-nsp12 plasmids into 293T cells. After 36 h, the supernatant was removed, and immunoprecipitation was performed using EGFP-specific magnetic beads. **B** Co-transfection of cells with HA-Ub mutant plasmids, Flag-RNF187, and EGFP-nsp12 plasmids into 293T cells. After 36 h, the supernatant was removed, and immunoprecipitation was performed using EGFP-specific magnetic beads. **C** Co-transfection of cells with Flag-nsp12 mutant plasmids and HA-RNF187 plasmids into 293T cells. After 36 h, the supernatant was removed, and western blotting was performed. **D** HEK293 cells were co-transfected with HA-K63 ubiquitin, Flag-nsp12^K130A^ and Myc-RNF187 as indicated. Cell lysates were immunoprecipitated with anti-Flag-specific magnetic beads. Whole-cell lysates (WCL) were used to verify the protein expression levels.

The selective autophagy pathway involves selective autophagy receptor-mediated recognition of ubiquitinated substrates, which are then delivered to autophagosomes for degradation via the selective autophagy pathway [19]. To verify whether RNF187 promoted nsp12 ubiquitination, we performed immunoprecipitation experiments. HA-Ub, Flag-RNF187, and EGFP-nsp12 were co-transfected into HEK-293T cells. RNF187 induced nsp12 ubiquitination (Figure 6A). To determine which type of polyubiquitination led to nsp12 ubiquitination, we co-transfected seven ubiquitin mutant plasmids with Flag-RNF187 and EGFP-nsp12 into HEK-293T cells for ubiquitination detection. Only the K63-linked ubiquitination of nsp12 was increased (Figure 6B). Subsequently, six lysine sites of nsp12 were mutated and the corresponding plasmids were constructed. These mutant plasmids were co-transfected with RNF187 into cells for protein detection. The degradation of nsp12 by RNF187 was reversed only when the K130 site was mutated (Figure 6C). Subsequently, co-immunoprecipitation results showed that overexpression of RNF187 significantly reduced the K63-linked ubiquitination of nsp12^K130A^ compared with the control group (Figure 6D). These findings indicate that RNF187 targets the K130 site of nsp12 for K63 ubiquitination.

## Discussion

PRRSV continues to pose challenges to swine farmers owing to its significant genetic diversity, macrophage tropism, persistent infection, environmental persistence, and ability to evade host immune responses [20]. Nsp12, as a key component of viral RNA synthesis, regulates viral replication by interacting with the host proteins. For example, the proteasome subunit PSMA2 has been identified as a novel host-restriction factor against PRRSV. PSMA2 inhibits PRRSV replication by enhancing proteasome and immunoproteasome activities. The PRRSV nsp12 mediates the specific degradation of PSMA2 via the autophagic pathway [21]. Conversely, the E3 ubiquitin ligase RNF114 interacts with nsp12 and promotes its degradation via K27-linked polyubiquitination, thereby significantly inhibiting PRRSV replication [10]. Galectin-3-binding protein (LGALS3BP) mediates ubiquitination of the nsp12 at lysine residue 91 by recruiting Cullin3 E3 ubiquitin ligase through its BACK domain, leading to proteasomal degradation. This process disrupts virus subgenomic RNA synthesis dependent on nsp12, thereby inhibiting PRRSV replication. Additionally, LGALS3BP enhances antiviral innate immunity by upregulating interferon-β (IFN-β) and interferon-stimulated genes [22]. Galectin-3 inhibits PRRSV replication by interacting with the viral nsp12 in vitro [12]. The proteasome component PSMB1 interacts with viral nsp12 and mediates autophagic degradation. Concurrently, PSMB1 exerts anti-PRRSV activity by interacting with the selective cargo receptor protein NBR1 and the E3 ubiquitin ligase STIP1 homology and U-box containing protein 1 (STUB1) [11]. These studies indicate that nsp12 can interact with multiple host proteins to regulate viral replication; however, the specific molecular mechanisms underlying this process remain unclear. Therefore, this study identified the host protein RNF187, which interacts with nsp12, using yeast two-hybrid technology, and further investigated the role of RNF187 in PRRSV infection.

Host cells can utilize multiple host-restriction factors to target the degradation of key PRRSV viral proteins through two major core pathways: the ubiquitin–proteasome system (UPS) and the lysosomal pathway, thereby inhibiting viral replication and constituting an important component of the host innate antiviral defense system [23]. This study confirmed that the E3 ubiquitin ligase RNF187 exerts a significant antiviral effect during PRRSV infection. Through overexpression and knockdown experiments, we found that RNF187 overexpression significantly reduced the viral titer and viral N protein expression, whereas RNF187 knockdown promoted viral replication (Figure 3). This is similar to other host antiviral factors identified in recent years. For example, NLR family, pyrin domain containing 12 (NLRP12) can bind to PRRSV GP2a via its LRR domain, recruit the E3 ligase membrane-associated RING-CH finger 8 (MARCH8) via its PYD domain, catalyze the K48-linked ubiquitination of GP2a at the K128 site, and subsequently mediate its degradation through the MARCH8-NDP52 pathway, thereby blocking viral assembly and infection [24]. Cholesterol 25-hydroxylase (CH25H) can mediate the ubiquitination of viral nsp1 at the K169 site and its subsequent proteasomal degradation, in a manner either dependent on or independent of its enzymatic activity. Additionally, it inhibits viral entry through the product 25-hydroxycholesterol [25].

Many studies have demonstrated that PRRSV can hijack components of the host UPS, particularly E3 ubiquitin ligases, to favor its replication and immune evasion. On one hand, PRRSV viral proteins can act as “molecular baits” to recruit host E3 ligases, thereby degrading key host antiviral proteins (such as RIG-I, MDA5, and IRF3), inhibiting IFN production, and achieving immune evasion. On the other hand, viral proteins can also be targeted for ubiquitination and degradation by certain host E3 ligases, which restricts viral replication. Some E3 ligases are exploited by the virus to promote replication, whereas others are utilized by the host to restrict the virus and maintain antiviral homeostasis [26]. Previous studies have reported that tripartite motif containing 28 (TRIM28) interacts with PRRSV GP4 and reduces its K63-linked polyubiquitination, thereby increasing protein expression, maintaining viral protein stability, and facilitating viral assembly and release [27]. Meanwhile, RNF122 catalyzes the K63-linked ubiquitination of PRRSV nsp4, enhancing nsp4 protein stability and promoting the function of the viral replication complex and viral RNA synthesis. Additionally, RNF122 performs K27- and K48-linked ubiquitination of MDA5, degrades MDA5, and inhibits IFN production, ultimately promoting viral proliferation [28]. In contrast, TRIM29 directly interacts with nsp11 and exerts E3 ligase activity to catalyze K48-linked ubiquitination. TRIM29 significantly reduced nsp11 protein levels, thereby impairing viral replication [29]. PRRSV N protein interferes with the interaction between TRIM25 and RIG-I by competitively interacting with TRIM25. Additionally, the N protein inhibits TRIM25-mediated ubiquitination of RIG-I expression to suppress IFN-β production, thereby inhibiting PRRSV replication [30]. TRIM25 also interacts with KEAP1 and Nrf2, targeting KEAP1 for K48-linked ubiquitination and proteasomal degradation, promoting the nuclear translocation of Nrf2, and activating Nrf2-mediated p62 expression. This further inhibits autophagy mediated by the KEAP1-Nrf2-p62 axis, ultimately suppressing PRRSV replication [31]. Therefore, host E3 ubiquitin ligases play a dual role in PRRSV infection, and in our study, we found that RNF187 can induce autophagic flux. Furthermore, RNF187 degraded nsp12 via the autophagic pathway, thereby inhibiting PRRSV replication (Figures 3, 5).

Previous studies have demonstrated that RNF187 directly interacts with the p53 protein encoded by the tumor suppressor gene TP53. RNF187 catalyzes the K48-linked polyubiquitination of the p53 protein, promoting its degradation via the proteasome and thereby inhibiting the activation of the p53 signaling pathway, which affects cellular growth and apoptotic processes [16]. In inflammatory responses, RNF187 is a homodimer of c-Jun that can specifically bind to c-Jun and promote c-Jun/activating protein 1 (AP-1)-dependent gene transcriptional activity [32]. In the mouse spermatogonia cell line GC-1, the E3 ubiquitin ligase RNF187 significantly promotes the degradation of KRT36/KRT84 via lysine 48-linked polyubiquitination. This degradation increases the expression of Notch signaling pathway-related genes, which enhance GC-1 cell proliferation and migration while suppressing apoptosis [33]. RNF187 interacts with H3 and directly or indirectly mediates its ubiquitination of H3 at lysine 57 (K57) or lysine 80 (K80), resulting in increased cellular transcription [34]. However, its interactions with viruses have not yet been reported. In this study, RNF187 degraded nsp12 through an autophagic pathway rather than through the classical ubiquitin–proteasome pathway, thereby expanding the functional patterns of E3 ubiquitin ligases in antiviral innate immunity. Selective autophagy is a potent strategy for host antiviral immunity, and selective autophagy cargo receptors include p62/SQSTM1, NBR1, NDP52, TOLLIP, and OPTN. Cargo receptors are crucial for the selection and transport during selective autophagy. In this study, we confirmed that NDP52 is the cargo receptor responsible for recruiting nsp12 for degradation (Figure 5). We also demonstrated that ubiquitination occurs at the K130 site of nsp12 (Figure 6). However, the pathogenicity of the mutant strain with this site mutation was not investigated in this study, which may represent a potential target for future antiviral drug development.

**Figure 7:**
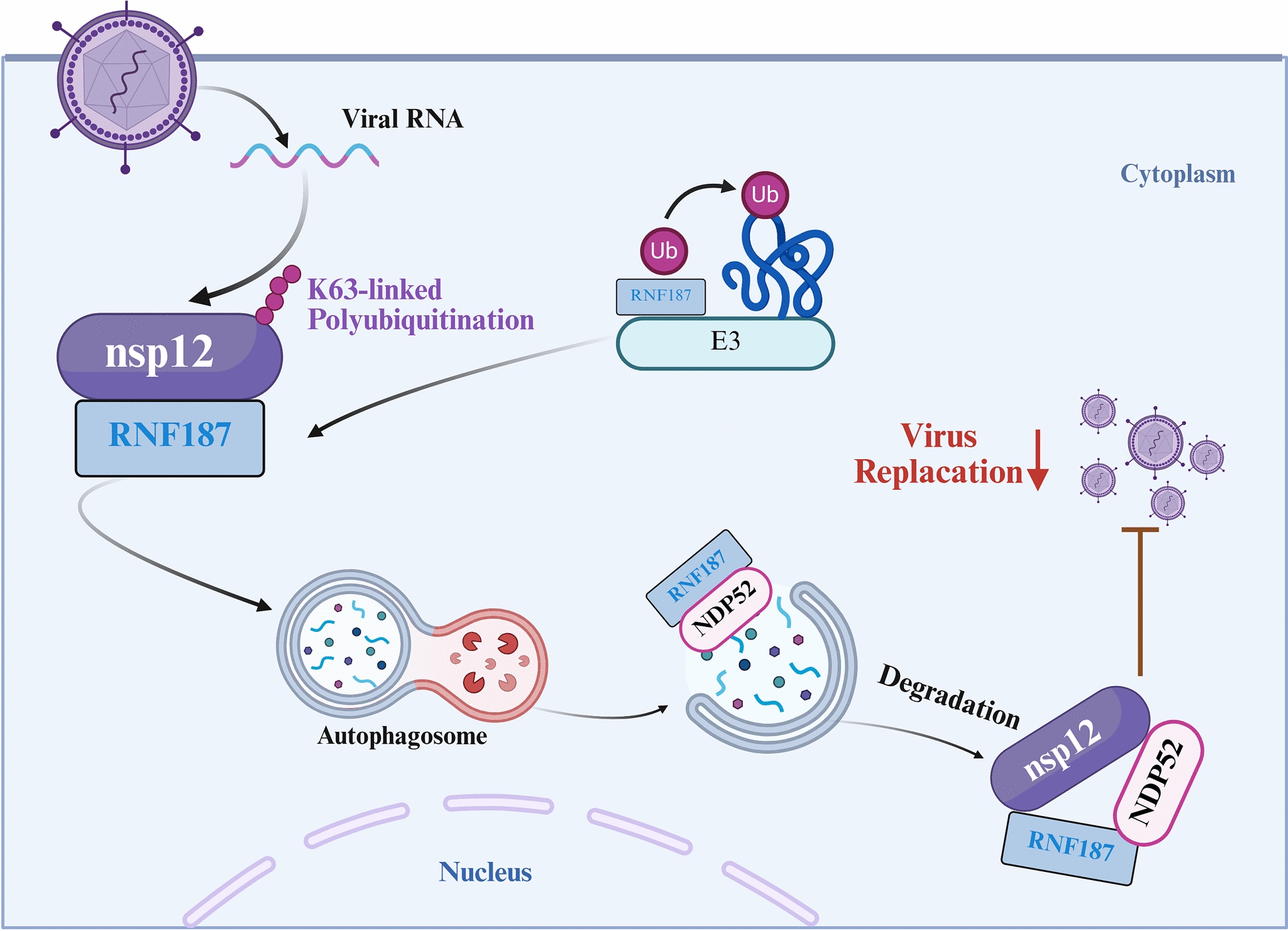
**Nsp12-RNF187 interaction model.** After PRRSV infection, the host protein RNF187 targets nsp12 for degradation via the autophagic pathway. The K130 site of nsp12 is a critical site for this process. RNF187 mediates the degradation of nsp12 by recruiting the autophagic receptor NDP52, thereby inhibiting viral replication.

In this study, we demonstrated that RNF187 mediates the autophagic degradation of nsp12 via ubiquitination at the K130 site through interaction with the selective autophagy cargo receptor, NDP52, thereby exerting anti-PRRSV effects (Figure 7). Our research revealed the key role of host-virus protein interaction mechanisms in the pathogenesis of PRRSV.

## Data Availability

No datasets were generated or analyzed during the current study.
